# Inflammatory myopathy and severe rhabdomyolysis induced by leuprolide acetate therapy for prostate cancer: a case report

**DOI:** 10.1186/1752-1947-5-409

**Published:** 2011-08-24

**Authors:** Michael Bergner, Martin Rohacek, Paul Erne

**Affiliations:** 1 Department of Cardiology, Luzerner Kantonsspital, 6000 Luzern 16, Switzerland; 2 Emergency Department, University Hospital Bern, 3010 Bern, Switzerland

## Abstract

**Introduction:**

Leuprolide acetate is a synthetic analog of gonadotropin-releasing hormone used for the treatment of prostate cancer. Its side effects are hot flashes, nausea, and fatigue. We report a case of a patient with proximal inflammatory myopathy accompanied by severe rhabdomyolysis and renal failure following the second application of leuprolide acetate. Drug withdrawal and steroid therapy resulted in remission within six weeks of the diagnosis. To the best of our knowledge, our case report describes the second case of leuprolide acetate-induced inflammatory myopathy and the first case of severe leuprolide acetate-induced rhabdomyolysis and renal failure in the literature.

**Case presentation:**

A 64-year-old Swiss Caucasian man was admitted to the hospital because of progressive proximal muscle weakness, dyspnea, and oliguria. He had been treated twice with leuprolide acetate in monthly doses. We performed a muscle biopsy, which excluded other causes of myopathy. The patient's renal failure and rhabdomyolysis were treated with rehydration and steroid therapy.

**Conclusion:**

The aim of our case report is to highlight the rare but severe side effects associated with leuprolide acetate therapy used to treat patients with inflammatory myopathy: severe rhabdomyolysis and renal failure.

## Introduction

The etiology of myopathy includes congenital disorders, immunologic processes, malignancies, infections, endocrinopathies, alcohol ingestion and adverse drug reactions (particularly statins), immunosuppressive agents, and nucleoside analog reverse transcriptase inhibitors [1-5]. Drugs can exert myotoxic effects on muscles through mechanisms that are direct (for example, alcohol ingestion, statins, or anti-malarial agents), immunological (for example, interferon α), or indirect (for example, drug-induced hypokalemia, hyperthermia, or seizures). Myositis can be associated with different cancers, mainly lung and breast cancers, but also prostate cancer. In one study, cancer was diagnosed in 9% of 396 patients with polymyositis, and of the 168 men with polymyositis, four had prostate cancer [6]. In another study, of 309 patients with dermatomyositis or polymyositis, 11.9% had cancer and one of these had prostate cancer [7]. Myopathy might affect all muscles or only proximal muscles, as well as pharyngeal muscles.

## Case report

**Figure 1:**
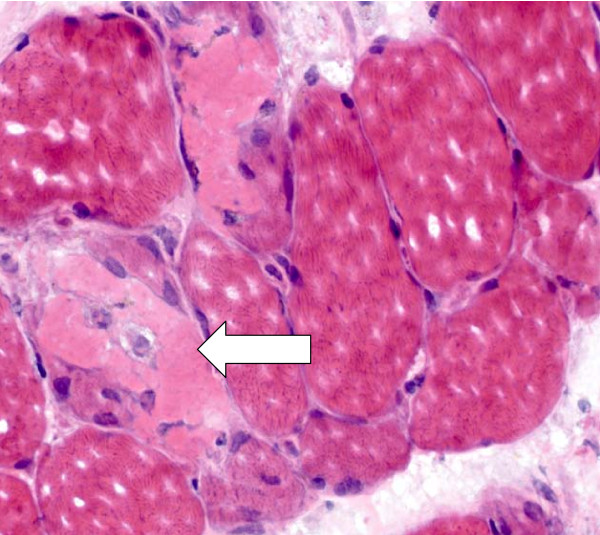
**Cross-sectional slice of human skeletal muscle showing acute muscle fiber necrosis (hematoxylin and eosin stain; × 400 original magnification)**.

**Figure 2:**
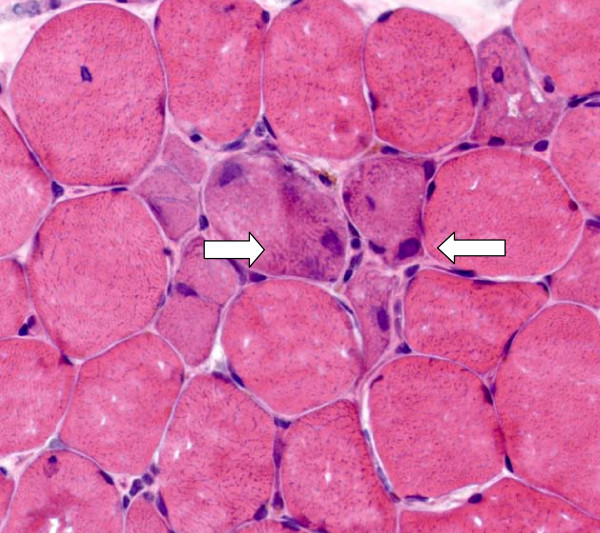
**Cross-sectional slice of human skeletal muscle (hematoxylin and eosin stain; × 400 original magnification)**. Regenerating muscle fibers can be seen as basophilic fibers with enlarged nuclei in the center of the image.

**Figure 3:**
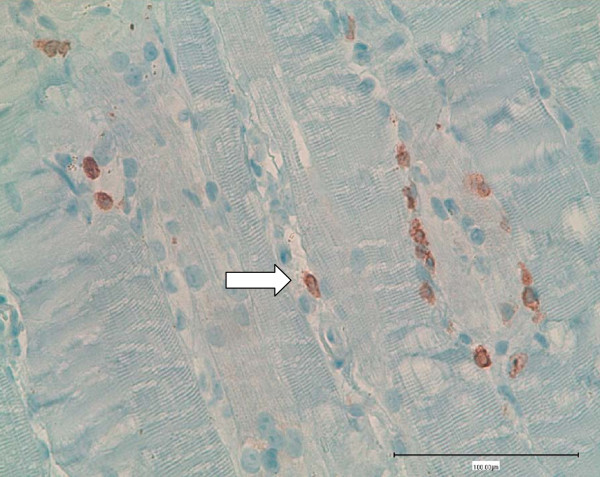
**Immunohistochemical staining for CD3 to display T lymphocytes**.

A 64-year-old Swiss Caucasian man patient with weak urinary flow and an elevated serum prostate-specific antigen (PSA) level of 130 μg/L was diagnosed with adenocarcinoma of the prostate on the basis of a biopsy (Gleason grade G3, Gleason score 4 + 4 = 8). A chest X-ray obtained for further staging showed a solitary node 9 mm in size in the left lower lung lobe. A computed tomographic scan of the patient's abdomen and skeletal nuclear scintigraphy revealed no further suspicious malignancies. Leuprolide acetate therapy delivered as a monthly dose was initiated. After two months of therapy, his serum PSA level decreased to 7 μg/L, and a chest X-ray showed complete regression of the lung node. A second dose of leuprolide acetate delivered every three months was applied. Two months later the patient was admitted to the hospital because of progressive proximal muscle weakness of six weeks' duration; slight, intermittent proximal muscle pain; dyspnea; and oliguria. He was treated with irbesartan and hydrochlorothiazide (CoAprovel^® ^150/12.5 mg Sanofi Pharma Bristol - Myers Squibb SNC, 174 Avenue de France F - 75013 Paris, France) because of arterial hypertension and tamsulosin (Pradif T^® ^Boehringer Ingelheim GmbH, Dufourstrasse 54 CH 4002 Basel, Switzerland) because of weak urinary flow. He did not drink alcohol but smoked one pack of cigarettes per day. At the time of admission, the patient was alert, his body temperature was 38.6°C, his blood pressure was 140/80 mmHg, his heart rate was 80 beats/minute, his breathing rate was 20 breaths/minute, and his oxygen saturation level was 85% while breathing ambient air. He had edema in his lower legs. Painless muscle weakness prevented him from standing or sitting. He had normal strength in both his hands and his feet, but active lifting of his head, legs, and arms was barely possible while he was supine, and his speech was slurred. His reflexes, eye movements, and cranial nerve function were normal. He had no skin lesions. A chest X-ray showed right lung infiltration consistent with aspiration pneumonia. No signs of lung fibrosis were observed. His electrocardiogram was normal. His laboratory values were as follows: hemoglobin 148 g/L, leukocyte count 14.7 × 10^9^/L, erythrocyte sedimentation rate 14 mm/hour, creatine kinase 121,530 U/L, C-reactive protein 39 mg/L, creatinine 51 μmol/L, BUN 6.4 mmol/L, sodium 122 mmol/L, and potassium 4.3 mmol/L. His serum 25-OH vitamin D level and thyroid gland function were normal, and his human immunodeficiency virus test was negative. MRI of his legs showed edema of the proximal muscles, particularly of both adductors. A biopsy of adductor muscle tissue was performed. Histological and immunohistochemical tests (inflammation marker, membrane attack complex, and major histocompatibility complex class I) showed signs of muscle necrosis (Figure 1, Figure 2) and diffuse muscle infiltration of T lymphocytes (Figure 3), but no signs of an autoimmune process. Additional serological tests for hepatitis B, hepatitis C, anti-nuclear antibodies, anti-neutrophil cytoplasmic antibodies, anti-double-stranded DNA antibodies, anti-mitochondrial M2 antibodies (anti-Mi2), anti-signal recognition particle (anti-SRP) antibodies, anti-Jo-1 antibodies and anti-polymyositis/scleroderma antibodies (anti-PM-Scl) all yielded negative results.

The patient was rehydrated with bicarbonate solution until his jugular veins were distended, and therapy with intravenous furosemide, ceftriaxone, methylprednisolone (500 mg/day), calcium, vitamin D, and alendronate was initiated. The patient's proximal muscle weakness declined within three days. Within four days, his serum creatinine level rose to 190 μmol/L, which was accompanied by oliguria, and his serum creatine kinase level dropped from a maximum of 169,910 U/L to 34,897 U/L. His steroid therapy was modified to oral prednisone 80 mg/day. Seven days later he could walk again with support, and his urine output and serum creatinine level had normalized. After 28 days, his prednisone treatment was tapered back to 35 mg/day, but within four days his serum creatine kinase rose again from 547 U/L to 1548 U/L without clinical deterioration. His prednisone dosage was increased to 70 mg/day, and his serum creatine kinase declined to normal (246 U/L) within six weeks. The patient was discharged from the hospital free of symptoms after undergoing orchiectomy on the 45th day following his initial admission. His serum creatine kinase and serum creatinine were normal, and he was prescribed prednisone 50 mg/day. After his discharge from the hospital, prednisone was tapered to 20 mg/day and his serum creatine kinase level rose slightly without clinical relapse. Nine months after discharge his prednisone therapy was stopped without a subsequent increase in his creatine kinase level. At his 12-month follow-up examination, the patient was in good clinical condition and had normal laboratory values, including PSA.

## Discussion

Myopathy with rhabdomyolysis and renal failure can have several causes. In the course of searching for a possible inflammatory myopathy, we found no clinical or serological signs of endocrinopathies, viral infections, or connective tissue diseases and no immunohistochemical signs of autoimmune polymyositis or dermatomyositis. The negative results of screening of our patient for these antibodies represent an additional argument against a diagnosis of autoimmune polymyositis, although anti-Jo-1 antibodies are found in 18% to 55% of these patients, anti-Mi-2 antibodies are found in 4% to 9%, anti-SRP antibodies are found in 4.8% to 11% of patients with autoimmune polymyositis, and anti-PM-Scl antibodies are found in 25% of patients with concomitant polymyositis and scleroderma [8-10]. Although cancer-associated myopathy can be of inflammatory origin [11], we assume that a paraneoplastic etiology of the myositis in our patient is not probable. While cancer-associated myositis can ameliorate during cancer treatment [7], the treatment of cancer in our patient resulted in the development of clinical signs of myopathy. On the basis of his serum PSA and the fact that the size of the solitary node in his lung decreased during leuprolide acetate therapy, we assume that prostate cancer therapy was successful in our patient.

We consider leuprolide acetate to be the cause of myopathy in our patient. We did not find an infectious cause of his myopathy. However, T lymphocytes were found. Thus, it is likely that a drug-induced T-lymphocyte-mediated mechanism, not a direct toxic effect of leuprolide acetate, caused his muscle necrosis. We cannot explain the exact mechanism. Further studies are required to answer this question.

To the best of our knowledge, this is the fourth such case reported in the literature. The three previously published cases manifested with proximal myopathy. While the muscle biopsy in the first case showed diffuse T-lymphocyte infiltration of the muscle and was treated with steroids [12], the biopsy in the second case revealed only mild, non-specific changes [13]. In the third case, no biopsy was performed [14]. In these three studies, the reported serum creatine kinase values were 2728 U/L, normal, and 1290 U/L, respectively, and within one to six months after leuprolide acetate therapy was discontinued, the patients' symptoms of myopathy vanished. Renal failure did not occur.

## Conclusion

In this case report, we highlight rare but severe side effects of leuprolide acetate therapy: inflammatory myopathy, severe rhabdomyolysis, and renal failure. To the best of our knowledge, this report describes the second case of leuprolide acetate-induced inflammatory myopathy and the first case of severe leuprolide acetate-induced rhabdomyolysis with renal failure.

## Consent

Written informed consent was obtained from the patient for publication of this case report and any accompanying images. A copy of the written consent is available for review by the Editor-in-Chief of this journal.

## Competing interests

The authors declare that they have no competing interests.

## Authors' contributions

MB and MR contributed equally to the case report. Both of them wrote the report and conducted the literature search. PE contributed to the discussion and supervised the writing of the case report. All authors were involved with the treatment of the patient, and all authors read and approved the final manuscript.

## References

[B1] Klopstock T Drug induced myopathies Curr Opin Neurol 2008 21 590 595 10.1097/WCO.0b013e32830e2774 18769254

[B2] Buchbinder R Forbes A Hall S Denett X Giles G Incidence of malignant disease in biopsy-proven inflammatory myopathy Ann Intern Med 2001 134 1087 1095 11412048 10.7326/0003-4819-134-12-200106190-00008

[B3] Stockton D Doherty VR Brewster DH Risk of cancer in patients with dermatomyositis or polymyositis, and follow-up implications: a Scottish population-based cohort study Br J Cancer 2001 85 41 45 10.1054/bjoc.2001.1699 11437400 PMC2363903

[B4] Bannwarth B Drug-induced myopathies Expert Opin Drug Saf 2002 1 65 70 10.1517/14740338.1.1.65 12904161

[B5] Bannwarth B Drug-induced musculoskeletal disorders Drug Saf 2007 30 27 46 10.2165/00002018-200730010-00004 17194169

[B6] Sigurgeirsson B Lindelöf B Edhag O Allander E Risk of cancer in patients with dermatomyositis or polymyositis N Engl J Med 1992 326 363 367 10.1056/NEJM199202063260602 1729618

[B7] András C Ponyi A Constantin T Csiki Z Szekanecz E Szodoray P Dankó K Dermatomyositis and polymyositis associated with malignancy: a 21-year retrospective study J Rheumatol 2008 35 438 444 18203322

[B8] Targoff IN Autoantibodies and their significance in myositis Curr Rheumatol Rep 2008 10 333 340 10.1007/s11926-008-0053-2 18662515

[B9] Targoff IN Myositis specific autoantibodies Curr Rheumatol Rep 2006 8 196 203 10.1007/s11926-996-0025-3 16901077

[B10] Ghirardello A Zampieri S Tarricone E Iaccarino L Bendo R Briani C Rondinone R Sarzi-Puttini P Todesco S Doria A Clinical implications of autoantibody screening in patients with autoimmune myositis Autoimmunity 2006 39 217 221 10.1080/08916930600622645 16769655

[B11] Levine SM Cancer and myositis: new insights into an old association Curr Opin Rheumatol 2006 18 620 624 10.1097/01.bor.0000245721.02512.77 17053509

[B12] Crayton H Bohlmann T Sufit R Graziano FM Drug induced polymyositis secondary to leuprolide acetate (Lupron) therapy for prostate carcinoma Clin Exp Rheumatol 1991 9 525 528 1954704

[B13] Van Gerpen JA McKinley KL Leuprolide-induced myopathy J Am Geriatr Soc 2002 50 1746 1747 10.1046/j.1532-5415.2002.50474.x 12366635

[B14] Salvador Hernández J Ferrera Rodríguez R Martín Oliva MV LH-RH analogues and myopathy in Spanish Aten Primaria 2004 34 265 10.1157/13066407 15456576 PMC7668978

